# Overall Survival Following Interval Complete Gross Resection of Advanced Ovarian Cancer via Laparoscopy Versus Open Surgery: An Analysis of the National Cancer Database

**DOI:** 10.3390/jcm14041164

**Published:** 2025-02-11

**Authors:** Judy Hayek, Anjile An, Jennifer Wolf, Kelly Lamiman, Michael Kim, Hope Knochenhauer, Nicole Goncalves, Ioannis Alagkiozidis

**Affiliations:** 1 Department of Gynecologic Oncology, SUNY Downstate Health Sciences University, Brooklyn, NY 11203, USAklamiman@maimo.org (K.L.); ialagkiozidis@maimo.org (I.A.); 2 Department of Obstetrics and Gynecology, Maimonides Medical Center, Brooklyn, NY 11219, USA; ngoncalves@maimo.org; 3 Division of Biostatistics, Department of Population Health Sciences, Weil Cornell Medicine/NewYork-Presbyterian, New York, NY 10065, USA; jia4001@med.cornell.edu; 4 Department of Obstetrics and Gynecology, NewYork-Presbyterian, Brooklyn, NY 10065, USA; mjk2008@nyp.org; 5 Department of Obstetrics and Gynecology, Hofstra/Northwell at Staten Island University Hospital, Staten Island, NY 10305, USA; hknochenhaue@northwell.edu

**Keywords:** robotic surgery, interval debulking, ovarian cancer, R0 resection, minimally invasive surgery

## Abstract

**Background:** Advanced epithelial ovarian cancer (EOC) has traditionally been treated with primary debulking surgery; however, recent phase III trials have demonstrated similar survival outcomes for patients who were randomized to neoadjuvant chemotherapy followed by interval debulking (IDS) when compared to patients who underwent PDS. **Methods:** We sought to evaluate a cohort of patients with EOC diagnosed between 2010 and 2019 who underwent complete cytoreduction (R0—no gross residual disease) during IDS. We compared the outcomes after R0 resection via MIS versus laparotomy in IDS. The primary endpoint was overall survival (OS). Kaplan-Meier analysis and inverse probability of treatment weighting (IPTW) were used. Cases were stratified by surgical extent and within the MIS cohort by robotic assistance. Surgical outcomes (LOS, readmission rate, 30- and 90-day mortality) were also assessed. **Results:** In total, 2412 patients were eligible. 624 (25.8%) underwent R0 resection via MIS. Over the study period, the MIS utilization rate increased from 12% to 36%. There was no significant difference in OS between the MIS and open cohorts (51 vs. 46 months, HR 1.1; 95% CI 0.96–1.24). 30-day and 90-day postoperative mortality rates were higher in the open group (1.6% vs. 0.8%, *p* = 0.006) and (1.9% vs. 3.5%, *p* = 0.003), respectively. Patients in the MIS group were less likely to undergo extensive surgery (41% vs. 53%, *p* < 0.001). When stratified by surgical extent, no significant difference in OS was observed between MIS and laparotomy (49 vs. 44 months in the extensive surgery group and 53 vs. 50 months in the non-extensive surgery group). Within the MIS cohort, 49% of cases were performed robotically. OS did not differ significantly between robotic and conventional laparoscopic cases (52 vs. 50 months). From 2010 to 2019, there was an increase in the use of robot-assisted laparoscopy (from 6.2% to 25.5%), coinciding with a decline in the laparotomy rate (from 88.1% to 63.5%) (*p* = 0.008). **Conclusions:** R0 resection via MIS during IDS showed similar OS and decreased postoperative mortality compared to laparotomy. The increasing utilization of robotic assistance is associated with a decrease in the laparotomy rate.

## 1. Introduction

Epithelial ovarian cancer is the most lethal gynecologic malignancy. The American Cancer Society estimated that in 2024, approximately 19,600 women would receive a new diagnosis of ovarian cancer, and 12,740 will die from the disease [1]. Primary debulking surgery has been the traditional approach to treat advanced ovarian cancer; however, recent phase III trials have demonstrated similar survival outcomes and lower postoperative complication rates with neoadjuvant chemotherapy followed by interval debulking when compared to primary debulking surgery [2,3,4].

There has been rising interest in exploring a minimally invasive approach to the treatment of ovarian cancer. The National Comprehensive Cancer Network (NCCN) has recognized the role of minimally invasive surgery for managing early-stage disease and performing interval cytoreductive surgery after neoadjuvant chemotherapy [5]. Recent evidence has suggested that a minimally invasive approach (MIS) to interval debulking surgery (IDS) is feasible and safe [6,7,8].

Prior studies using the National Cancer Database [9] have demonstrated similar survival and lower morbidity after laparoscopic IDS compared to laparotomy [8,10]. The analyses in these studies were performed with the intention of treating. Diagnostic laparoscopy, prior to debulking surgery, has been widely used to assess the respectability of the disease to R0 (no gross residual disease), regardless of the route of completion of the procedure (MIS vs. open). Consequently, cases initiated laparoscopically solely to assess the feasibility of cytoreduction would have been included in the MIS group, potentially confounding the results. However, the NCDB does not provide information on the surgeon’s intent when using laparoscopy prior to debulking, which could have led to unintentional selection bias that was unaccounted for. In addition, these studies included all patients regardless of residual disease status, whereas residual disease is associated with worse survival rates and may have a confounding effect on the results.

Potential limitations of MIS for interval debulking are the lack of haptics and suboptimal exposure of certain anatomic locations such as the lesser sac and upper abdominal retroperitoneum, which could lead to underestimation of the amount of residual disease. It is possible that a disease that may be palpable or visible at laparotomy may not be visualized during laparoscopy, resulting in unrecognized residual disease and, hence, potentially suboptimal cytoreduction and worse survival outcomes. Since the amount of residual disease after cytoreductive surgery is the main survival predictive factor in ovarian cancer, a study assessing the survival of patients who achieved complete gross resection [11] after minimally invasive surgery versus open is critical to establishing the oncologic safety of the MIS approach.

In this study, we sought to evaluate a large cohort of women who underwent R0 cytoreduction (no gross residual disease) during IDS. The analysis was based on the mode of completion of the surgery regardless of the intent of the approach to eliminate potential selection bias. We aimed to compare overall survival and surgical outcomes between MIS and open R0 interval cytoreduction. We also stratified the cases based on the extent of surgery to address potential confounding factors and based on the use of robotic assistance to provide deeper insights into the impact of robotic assistance on survival outcomes and its association with the laparotomy rate.

## 2. Materials and Methods

**Figure 1:**
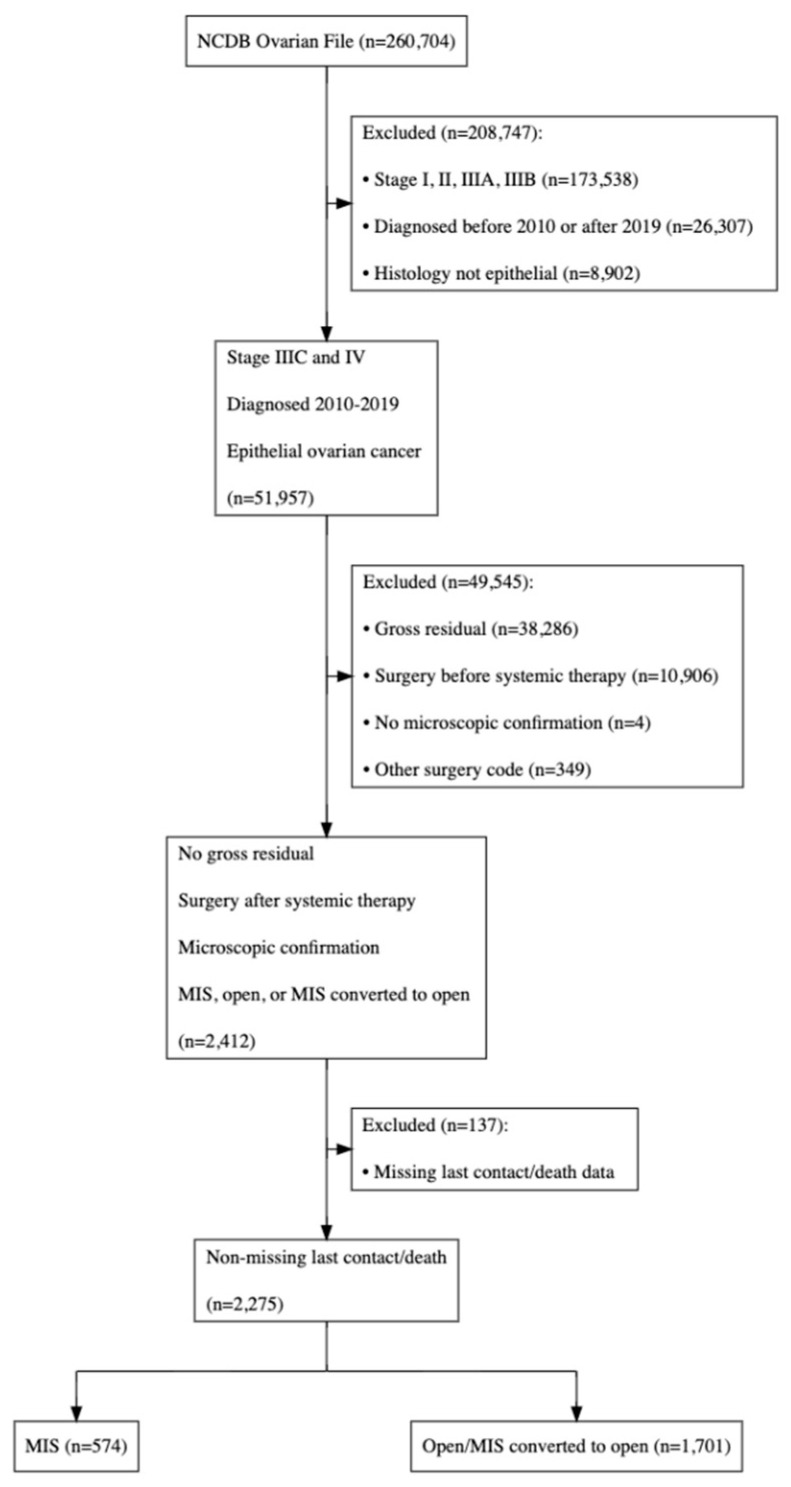
Selection and exclusion.

Data were extracted from the NCDB, a dataset on patients who received some element of their cancer care (treatment or diagnosis) at a cancer program that is accredited by the Commission on Cancer [12]—Accredited Centers. Data represents more than 70% of newly diagnosed cases collected in approximately 1500 facilities. The data used in this study were in an existing, deidentified database; therefore, exemption was granted by the Institutional Review Board at Maimonides Medical Center, Brooklyn, NY, USA. We identified a cohort of patients with stage IIIC and IV epithelial ovarian cancer diagnosed between January 2010 and December 2019 from the latest version available of the NCDB participant user data file provided by the NCDB. Clinical stage was used per the American Joint Commission on Cancer staging manual (7th edition) [13]. Given the survival disadvantage of residual disease after IDS, only patients who underwent complete surgical resection of gross disease as interval treatment of ovarian cancer after neoadjuvant chemotherapy were included in our analysis. Patients were then excluded in the setting of gross residual disease, surgery before systemic therapy, or no surgery/no systemic therapy and no microscopic confirmation (Figure 1). Serous, mucinous, endometrioid, clear cell and other adenocarcinoma were selected using the International Classification of Diseases for Oncology (3rd edition) [14]. The following variables were identified. Age was defined as a continuous variable in years. Race was categorized into White, Black, American Indian/Aleutian/Eskimo, Asian/Native Hawaiian/Pacific Islander, or other/unknown. Charlson–Deyo comorbidity scores (0–3) were used to measure comorbidity. The Charlson–Deyo value is a weighted score derived from the sum of the scores of each of the comorbid conditions. A value greater than 1 indicates the presence of comorbidities as defined by the ICD 9 or 10 secondary diagnosis codes. Facility type was subcategorized into community cancer program, comprehensive community cancer program, academic/research program, integrated network cancer program, or not available, a classification provided by the CoC. Rural-urban status was categorized as metropolitan, urban, rural, or unknown based on data published by the Department of Agriculture Economic Research Services (Washington, DC, USA) identifying patients’ state and county codes at the time of diagnosis.

Our analysis involved categorizing the cohort into two groups: the open group, which comprised surgeries conducted via laparotomy (planned or after MIS conversion), and the MIS group, which included all cases completed via laparoscopy or robotically (Figure 1).

The primary endpoint was overall survival, defined as the time from diagnosis to death or last contact. The secondary endpoint was the comparative length of postoperative hospitalization, unplanned readmission rate, and 30-day/90-day mortality rates between women who underwent MIS and open complete interval debulking.

The cases were further stratified based on the extent of surgery and the use of robotic assistance. We performed survival comparisons within the subgroups of non-extensive surgery defined as hysterectomy and/or oophorectomy +/− omentectomy and extensive surgery, which includes surgery to the bowel, urinary tract, and other organs, or radical surgery. The impact of robotic assistance on survival and its association with the rate of laparotomy was also evaluated. The missing data for the variables that affect survival were very few, and therefore, a sensitivity analysis was not deemed necessary.

### Statistical Analysis

Descriptive statistics were used to characterize the study cohort. Categorical variables are presented as frequency (%), and continuous variables are presented as mean (standard deviation). The Chi-squared test and Welch two-sample *t*-test were used to examine the association between demographic and clinical variables of interest and MIS/open surgery. The Chi-squared test was also used to compare the number of conversions to laparotomy based on a minimally invasive approach (laparoscopic vs. robotic). Kaplan–Meier survival analysis was used to assess overall survival (OS), and the log-rank test was performed to compare survival between MIS/open groups, laparoscopic/robotic groups, and extent of surgery groups. Multivariable Cox proportional hazards regression was performed to assess the independent effect of MIS/open surgery on OS. Inverse probability of treatment weighting (IPTW) with robust standard errors was used to balance the MIS/open surgery groups based on age, race, Charlson–Deyo comorbidity score, histology type, stage, and extent of surgery for a Cox proportional hazards regression model. (Table 1a,b) All *p*-values are two-sided with statistical significance evaluated at the 0.05 alpha level. All analyses were performed in R Version 4.3.1 (R Foundation for Statistical Computing, Vienna, Austria).

## 3. Results

A total of 2412 patients with stage IIIC and IV who underwent NACT followed by IDS with no residual disease met the eligibility criteria. 624 (25.9%) cases were completed via MIS, and 1788 (74.1%) were completed via open surgery (planned or after conversion from MIS). Robotic assistance was used in 49% of the cases that were completed via MIS. The proportion of cases that were completed via MIS increased from 11.8% in 2010 to 36.5% in 2019.

### 3.1. Patient Demographic, Clinical, and Tumor Characteristics

Compared with patients undergoing laparotomy, those undergoing MIS were older (66 years vs. 64 years, *p* < 0.016), more likely to have surgery in a comprehensive community cancer center (40% vs. 31%, *p* = 0.003), and in a metropolitan area (83% vs. 79%, *p* = 0.038). (Table 2) There was no difference between MIS and open surgery in terms of race, Charlson–Deyo comorbidity score, histology, or stage. Extensive surgery was more common in the open compared to the MIS cohort (53% vs. 41%, *p* < 0.001) (Table 2).

### 3.2. Survival Analysis

**Figure 2:**
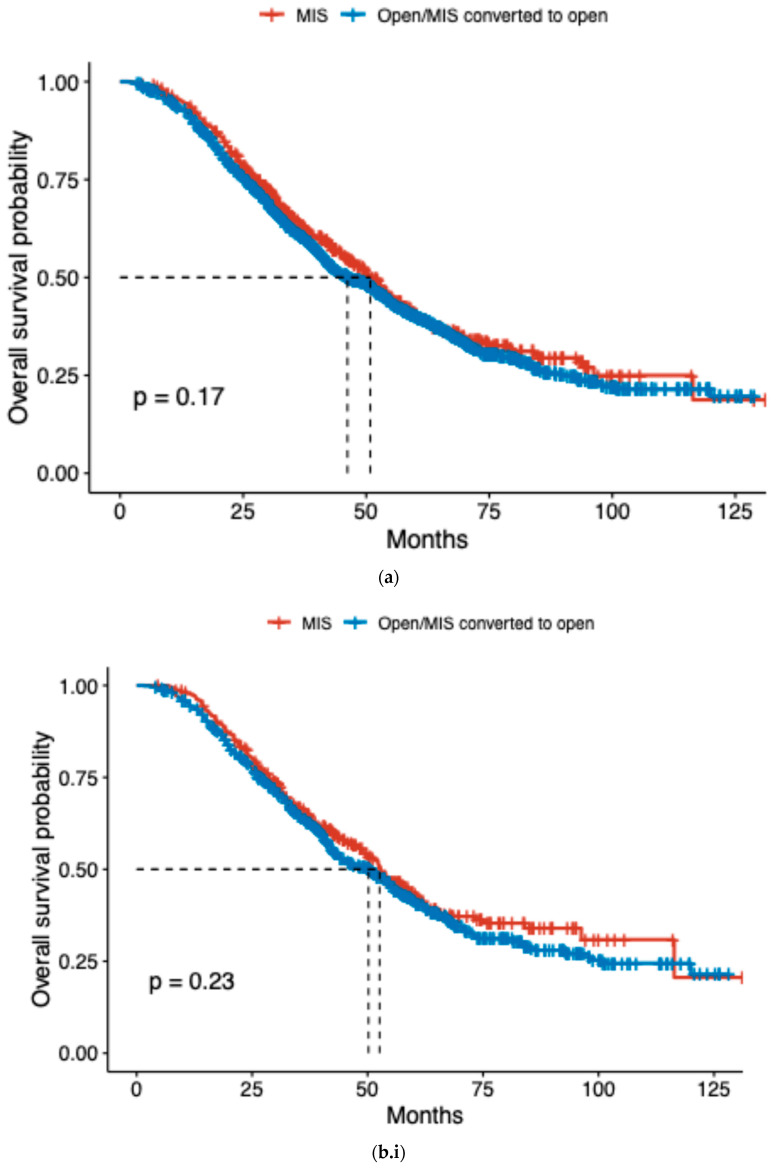

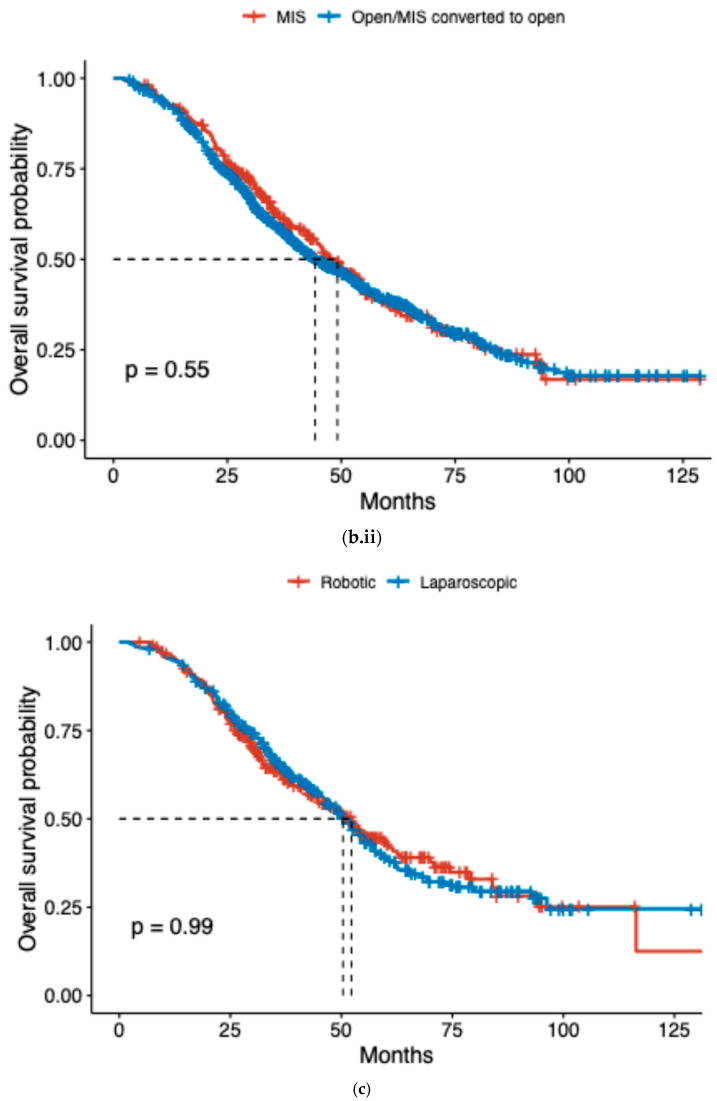
(**a**) Kaplan–Meier overall survival curves; (**b.i**) Non-Extensive Surgery Cohort; (**b.ii**) Extensive Surgery Cohort; (**c**) Kaplan–Meier overall survival curves stratified by minimally invasive approach.

Figure 2a shows the Kaplan-Meier survival comparison between MIS and open cases. The median overall survival for cases that were completed via open/converted to the open group was 46 months vs. 51 months in the MIS group (IPTW HR 1.10, 95% CI 0.94–1.26). In the multivariable Cox regression, factors associated with worse survival were older age (HR 1.01, 95% CI 1.01, 1.02), stage IV disease (HR 1.17, 95% CI 1.05, 1.30), and extensive surgery (HR 1.12, 95% CI 1.01, 1.25) (Table 3). In the group that had non-extensive surgery (hysterectomy and/or oophorectomy +/− omentectomy), there was no difference in OS between cases that were completed MIS vs. open (53 vs. 50 months, *p* = 0.23). (Figure 2b.i) Similarly, there was no difference in OS between MIS and open in the extensive surgery group (49 vs. 44 months, *p* = 0.55) (Figure 2b.ii).

### 3.3. Secondary Outcomes

As for secondary endpoints, mean length of stay was significantly shorter for the MIS group (3.3 vs. 5.3 days, *p* < 0.001). The median LOS decreased from 4 days in 2010 to 1 day in 2019 or MIS. There was no statistically significant difference in unplanned readmission rates between the two groups (2.1% vs. 2%, *p* = 0.50). Thirty-day mortality and 90-day mortality rates were higher in the open cohort (1.6% vs. 0.8%, *p* = 0.006) and (1.9% vs. 3.5%, *p* = 0.003), respectively (Table 4). When stratified to only examine secondary outcomes within the extensive surgery group, the mean length of stay was significantly shorter for the MIS group (4.2 vs. 6.1 days, *p* < 0.001). Readmission rates were similar between MIS and open surgery for this sub-cohort (4.3% vs. 4.3%, *p* = 0.98). Similarly, there was no statistically significant difference in 30-day and 90-day mortality between the two groups (1.6% vs. 1.9%, *p* = 0.92) and (3.5% vs. 4.2%, *p* = 0.85), respectively (Table 5).

### 3.4. Association of Robotic Assistance with Survival and Laparotomy Rate

When stratified by robotic versus conventional laparoscopic surgery within the MIS group, the log-rank test showed no difference in OS, with a median OS for the laparoscopic approach of 50 months vs. 52 months for the robotic approach (Figure 2c).

**Figure 3:**
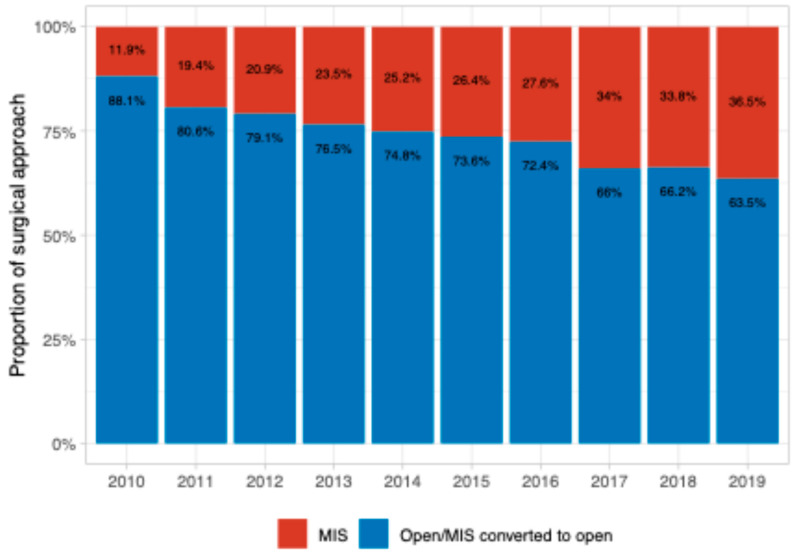
Proportion of MIS and open cases by year of diagnosis.

**Figure 4:**
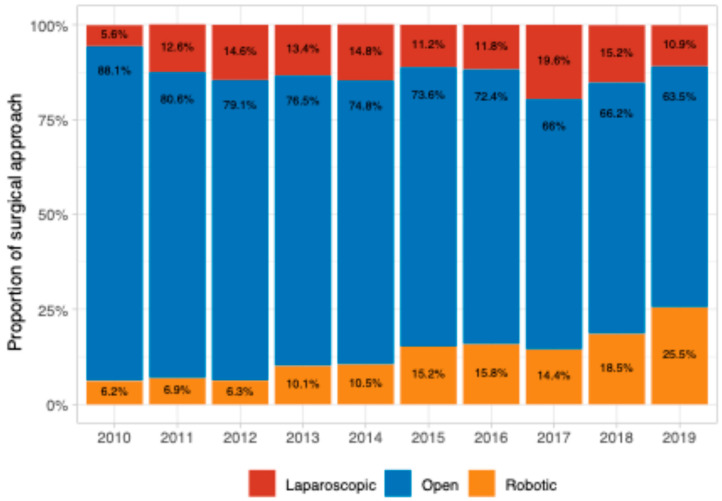
Proportion of robotic, laparoscopic, and open cases by year of diagnosis.

We further evaluated the temporal trends in the utilization of robot-assisted laparoscopy, conventional laparoscopy, and laparotomy. From 2010 to 2019, there was a decrease in the laparotomy rate (from 88.1% to 63.5%) (Figure 3) associated with the rising use of robot-assisted laparoscopy (increasing from 6.2% to 25.5%) (Figure 4 and Supplementary Table S2) (*p* = 0.008). However, there was no significant change in the proportion of cases completed via conventional laparoscopy throughout the study period (Figure 4). Of the 681 cases that were initiated MIS, 57 were converted to open for a conversion proportion of 8.3%. Out of the 371 cases that were initiated laparoscopically, 51 (13.7%) were converted to open, while out of the 310 cases that were initiated robotically, conversion to laparotomy occurred only in 6 (1.9%) (*p* < 0.001) (Supplementary Table S1).

## 4. Discussion

In this study, using a large cohort from the NCDB, we compared the survival outcomes of patients who had complete gross resection via MIS to those who had complete gross resection via laparotomy. We found no difference in overall survival but higher 30-day and 90-day mortality after open surgery. The length of stay was shorter for the MIS group.

The guidelines for the use of MIS for IDS remain vague; however, the NCCN has recognized its role in “select patients” [5]. The MISSION trial, a phase II trial, demonstrated the safety and feasibility of a minimally invasive approach for IDS in a well-selected population [6]. Other retrospective studies support this evidence [7,8].

As suggested by Fagotti et al., the utilization of a laparoscopic scoring system, namely the “Fagotti Score” to assess disease respectability has proven to be reliable [16,17]. If we take it a step further, diagnostic laparoscopy may also be a valuable tool to assess for laparoscopic respectability. However, limitations of MIS in debulking surgery include the lack of tactile feedback and suboptimal exposure, which could, in turn, lead to unrecognized residual disease and subsequently worse survival outcomes [5]. Nevertheless, the role of laparoscopy has been explored in upper abdominal surgery for ovarian cancer. Tozzi et al. have demonstrated the safety of the laparoscopic approach to diaphragmatic peritonectomy [18]. Furthermore, reduced perioperative morbidity and mortality associated with MIS could have a positive impact on overall survival. In a preclinical model of ovarian cancer, laparoscopy was associated with decreased surgical stress and attenuated tumor growth [19].

Prior retrospective observational studies show similar survival and improved perioperative outcomes with MIS but emphasize the surgeon’s role in selecting patients who are thought to be better candidates for an R0 resection [8,20,21,22]. This highlights the selection bias in the physician’s choice of IDS approach. Our study showed higher MIS rates in older patients, those treated at a comprehensive cancer community center in metropolitan areas, with no difference seen in terms of race, comorbidity scores, or stage. Therefore, the availability of resources, surgeon expertise, and patient-specific criteria affect the choice to perform minimally invasive IDS.

In our study, patients who underwent laparotomy were more likely to undergo extensive surgery (cytoreductive surgery, including radical hysterectomy and surgery to the bowel, bladder, and other organs). However, more extensive surgery did not offer a survival advantage, which likely reflects a higher disease burden in the laparotomy group. To overcome this potential selection bias, we stratified the cases based on the extent of surgery and found no survival difference between MIS and laparotomy in the two subgroups (extensive and non-extensive surgery). In fact, we also assessed perioperative outcomes after extensive cytoreduction, defined above, comparing MIS vs. laparotomy; while LOS was shorter with minimally invasive extensive surgery compared to open extensive surgery, readmission rates, and mortality rates remained the same.

Our data provide deeper insight into the impact of robotic surgery on outcomes. While we did not find a significant difference in overall survival between robotic and conventional laparoscopic cases, the temporal trends of the use of different surgical approaches reveal a decline in laparotomy rates over time, which corresponds to an increase in the use of robot assistance, while the proportion of cases completed via conventional laparoscopy remained stable. Published meta-analyses support the finding that robotic and traditional laparoscopies do not differ in overall survival [7,23]. The inverse correlation between the use of robotic assistance and the laparotomy rate may be related to well-known advantages of robotic surgery, such as better visualization, a wider range of motion, and easier manipulation. These advantages could affect the surgeon’s decision to opt for MIS and decrease the possibility of conversion to laparotomy. Besides the impact of the robotic approach, the surgeons’ progress along the learning curve during the study period could have contributed to the decline in the laparotomy rate.

A recent trial by Van Driel et al. showed longer survival rates in advanced ovarian cancer patients undergoing hyperthermic intraperitoneal chemotherapy [11] at the time of IDS. This population underwent open surgery, but in our practice, we often perform MIS+ HIPEC at IDS. To date, no trial has studied the outcomes of HIPEC at the time of minimally invasive IDS; however, a retrospective study by Morton et al. showed no difference in progression-free survival between patients undergoing laparotomy + HIPEC vs. MIS+ HIPEC at the time of IDS. Furthermore, there was no difference in R0 resection, ICU admission, estimated blood loss, operative time, adverse events, or use of vasopressors between the cohorts. The LOS was shorter for the MIS+HIPEC cohort. More randomized data are needed to further support this data.

The NCDB offers a large cohort of patients with an accurate representation of real-world practices and outcomes in the US. We believe that the strength of our study is that it is focused on assessing the oncologic outcomes of patients who had complete gross resection and excludes patients with residual disease in both groups, which is a major factor influencing survival outcomes. The use of IPTW to balance the two groups and the stratification of cases based on the extent of surgery helped eliminate potential biases. We also ensured that our two groups were selected based on the route of completion of the debulking rather than the initial intent, which is not reported in the NCDB.

On the other hand, we acknowledge several limitations to our study, including its retrospective nature. Data on patient-specific tumor burden, response to NACT, number of cycles, or choice of NACT were not available for analysis. Progression-free survival and cause of death were not included in the NCDB, which limited our ability to comment on them in our analysis. In addition, indications for conversion from MIS to laparotomy were not offered. Thus, it is unclear whether the extent to which the inverse correlation between the utilization of robotic assistance and the laparotomy rate is influenced by surgeon preference decreased likelihood of conversion, or the increasing availability of robotic assistance nationwide. Furthermore, the increased utility of the robotic approach over time may temporally overlap with new innovative treatments in ovarian cancer, such as the introduction of poly(ADP-ribose) polymerase (PARP) inhibitors, but we are unable to draw any conclusion given the lack of data on PARP inhibitor use in the NCDB.

In conclusion, this study demonstrates that R0 resection after minimally invasive IDS offers similar survival outcomes compared to R0 resection after open IDS, while the latter was associated with higher perioperative mortality. The increasing adoption of robot-assisted laparoscopy coincides with a decline in the laparotomy rate. While these data support the oncologic safety of MIS for IDS, we look forward to the results of a randomized prospective multicenter trial whose data may provide more solid grounds for the practice of minimally invasive interval debulking surgery [24].

## Figures and Tables

**Table 1:** (a). Balance before IPTW; (b). Balance after IPTW.

**(a)**					
**Variable**	**Open/MIS converted to open (Mean)**	**Open/MIS converted to open (SD)**	**MIS (Mean)**	**MIS (SD)**	**Diff Adj**
prop.score					
age	64.907	10.775	65.985	11.034	0.098
race_White	0.847	NA	0.855	NA	0.008
race_Black	0.091	NA	0.072	NA	−0.019
race_American Indian/Aleutian/Eskimo	0.005	NA	0.009	NA	0.004
race_Asian/Native Hawaiian/Pacific Islander	0.038	NA	0.042	NA	0.003
race_Other/Unknown	0.019	NA	0.022	NA	0.003
as.factor(cdcc_total_best)_0	0.797	NA	0.808	NA	0.011
as.factor(cdcc_total_best)_1	0.158	NA	0.151	NA	−0.008
as.factor(cdcc_total_best)_2	0.027	NA	0.025	NA	−0.002
as.factor(cdcc_total_best)_3	0.018	NA	0.017	NA	−0.001
histology_type_Serous carcinoma	0.784	NA	0.754	NA	−0.030
histology_type_Mucinous carcinoma	0.007	NA	0.007	NA	0.001
histology_type_Endometrioid carcinoma	0.016	NA	0.012	NA	−0.004
histology_type_Clear cell carcinoma	0.013	NA	0.008	NA	−0.005
histology_type_Other adenocarcinoma	0.181	NA	0.219	NA	0.038
stage_IV	0.530	NA	0.556	NA	0.026
extent_of_surgery_Extensive surgery	0.480	NA	0.373	NA	−0.107
extent_of_surgery_Gynecologic only/omentectomy	0.516	NA	0.624	NA	0.108
extent_of_surgery_Other/Unknown	0.004	NA	0.003	NA	−0.001
**(b)**					
**Variable**	**Open/MIS converted to open (Mean)**	**Open/MIS converted to open (SD)**	**MIS (Mean)**	**MIS (SD)**	**Diff Adj**
prop.score	0.263	0.052	0.263	0.054	0.008
age	65.418	10.644	65.460	11.117	0.004
race_White	0.849	NA	0.850	NA	0.002
race_Black	0.083	NA	0.082	NA	−0.001
race_American Indian/Aleutian/Eskimo	0.007	NA	0.007	NA	0.000
race_Asian/Native Hawaiian/Pacific Islander	0.040	NA	0.040	NA	0.000
race_Other/Unknown	0.021	NA	0.021	NA	−0.001
as.factor(cdcc_total_best)_0	0.805	NA	0.803	NA	−0.001
as.factor(cdcc_total_best)_1	0.152	NA	0.153	NA	0.001
as.factor(cdcc_total_best)_2	0.026	NA	0.026	NA	0.000
as.factor(cdcc_total_best)_3	0.017	NA	0.017	NA	0.000
histology_type_Serous carcinoma	0.770	NA	0.768	NA	−0.001
histology_type_Mucinous carcinoma	0.007	NA	0.007	NA	0.000
histology_type_Endometrioid carcinoma	0.014	NA	0.014	NA	0.000
histology_type_Clear cell carcinoma	0.010	NA	0.010	NA	0.000
histology_type_Other adenocarcinoma	0.199	NA	0.200	NA	0.002
stage_IV	0.539	NA	0.542	NA	0.003
extent_of_surgery_Extensive surgery	0.427	NA	0.425	NA	−0.002
extent_of_surgery_Gynecologic only/omentectomy	0.570	NA	0.571	NA	0.002
extent_of_surgery_Other/Unknown	0.003	NA	0.003	NA	0.000

**Table 2:** Patient characteristics by surgical approach.

	Overall N = 2412	MIS N = 624	Open/MIS Converted to Open N = 1788	*p* Value
**Age at diagnosis, years** [15]	65 (11)	66 (11)	64 (11)	**0.016**
**Race, n (%)**				0.56
White	2031 (84)	531 (85)	1500 (84)	
Black	233 (9.7)	52 (8.3)	181 (10)	
American Indian/Aleutian/Eskimo	13 (0.5)	5 (0.8)	8 (0.4)	
Asian/Native Hawaiian/Pacific Islander	92 (3.8)	24 (3.8)	68 (3.8)	
Unknown	43 (1.8)	12 (1.9)	31 (1.7)	
**Year of Diagnosis, n (%)**				**<0.001**
2010	177 (7.3)	21 (3.4)	156 (8.7)	
2011	175 (7.3)	34 (5.4)	141 (7.9)	
2012	253 (10%)	53 (8.5%)	200 (11%)	
2013	277 (11%)	65 (10%)	212 (12%)	
2014	305 (13%)	77 (12%)	228 (13%)	
2015	348 (14%)	92 (15%)	256 (14%)	
2016	304 (13%)	84 (13%)	220 (12%)	
2017	285 (12%)	97 (16%)	188 (11%)	
2018	151 (6.3%)	51 (8.2%)	100 (5.6%)	
2019	137 (5.7%)	50 (8.0%)	87 (4.9%)	
**Charlson–Deyo comorbidity score, n (%)**				0.62
0	1923 (80%)	509 (82%)	1414 (79%)	
1	378 (16%)	89 (14%)	289 (16%)	
2	67 (2.8%)	16 (2.6%)	51 (2.9%)	
3	44 (1.8%)	10 (1.6%)	34 (1.9%)	
**Facility Type, n (%)**				**0.003**
Community Cancer Program	33 (1.4%)	10 (1.6%)	23 (1.3%)	
Comprehensive Community Cancer Program	807 (33%)	248 (40%)	559 (31%)	
Academic/Research Program	1090 (45%)	250 (40%)	840 (47%)	
Integrated Network Cancer Program	437 (18%)	104 (17%)	333 (19%)	
Not Available	45 (1.9%)	12 (1.9%)	33 (1.8%)	
**Urban-Rural status, n (%)**				**0.038**
Metro	1933 (80%)	515 (83%)	1418 (79%)	
Urban	338 (14%)	84 (13%)	254 (14%)	
Rural	40 (1.7%)	11 (1.8%)	29 (1.6%)	
Not Available	101 (4.2%)	14 (2.2%)	87 (4.9%)	
**Histology Type, n (%)**				0.20
Serous carcinoma	1903 (79%)	483 (77%)	1420 (79%)	
Mucinous carcinoma	14 (0.6%)	4 (0.6%)	10 (0.6%)	
Endometrioid carcinoma	42 (1.7%)	8 (1.3%)	34 (1.9%)	
Clear cell carcinoma	37 (1.5%)	6 (1.0%)	31 (1.7%)	
Other adenocarcinoma	416 (17%)	123 (20%)	293 (16%)	
**Diagnostic Confirmation, n (%)**				0.66
Positive Cytology	140 (5.8%)	34 (5.4%)	106 (5.9%)	
Positive Histology	2272 (94%)	590 (95%)	1682 (94%)	
**Stage, n (%)**				0.65
IIIC	1186 (49%)	302 (48%)	884 (49%)	
IV	1226 (51%)	322 (52%)	904 (51%)	
**Extent of Surgery, n (%)**				**<0.001**
Extensive surgery	1206 (50%)	257 (41%)	949 (53%)	
Gynecologic only/omentectomy	1195 (50%)	365 (58%)	830 (46%)	
Other/Unknown	11 (0.5%)	2 (0.3%)	9 (0.5%)	

N: number.

**Table 3:** Multivariable Cox regression analysis of OS.

	HR	95% CI	*p*-Value
**Surgical Approach**			
MIS	—	—	—
Open/MIS converted to open	1.09	0.96, 1.24	0.18
**Age**	1.01	1.01, 1.02	<0.001
**Histology Type**			
Serous carcinoma	—	—	—
Mucinous carcinoma	1.61	0.86, 3.00	0.14
Endometrioid carcinoma	0.84	0.54, 1.30	0.43
Clear cell carcinoma	1.41	0.94, 2.12	0.10
Other adenocarcinoma	0.91	0.79, 1.05	0.18
**Stage**			
IIIC	—	—	—
IV	1.17	1.05, 1.30	0.005
**Charlson-Deyo Comorbidity Score**			
0	—	—	—
1	1.12	0.97, 1.29	0.11
2	1.22	0.90, 1.65	0.21
3	0.97	0.65, 1.44	0.87
**Extent of Surgery**			
Gynecologic only/omentectomy	—	—	—
Extensive surgery	1.12	1.01, 1.25	0.035
Other/Unknown	1.22	0.61, 2.46	0.57

**Table 4:** Perioperative outcomes according to the surgical approach.

	MIS N = 624	Open/MIS Converted to Open N = 1788	
Mean LOS [15]	3.3 (4.0)	5.3 (6.3)	**<0.001**
Readmission, n (%)	13 (2.1)	35 (2.0)	0.50
30-day Mortality, n (%)	5 (0.8)	28 (1.6)	**0.006**
90-day Mortality, n (%)	12 (1.9)	62 (3.5)	**0.003**

**Table 5:** Perioperative outcomes within the extensive surgery group.

	MIS N = 257	Open/MIS Converted to Open N = 949	
Mean LOS [15]	4.2 (5.2)	6.1 (7.9)	**<0.001**
Readmission, n (%)	11 (4.3)	41 (4.3)	0.98
30-day Mortality, n (%)	4 (1.6)	18 (1.9)	0.92
90-day Mortality, n (%)	9 (3.5)	40 (4.2)	0.85

## Data Availability

Data was available through National Cancer Database PUF user files applied for by the authors through the American College of Surgeons.

## Supplementary Materials

The following supporting information can be downloaded at: https://www.mdpi.com/article/10.3390/jcm14041164/s1, Table S1: Number of conversions to laparotomy based on minimally invasive approach; Table S2: The distribution of surgical approach per year.
