# Supplementary material for: Overall Survival Following Interval Complete Gross Resection of Advanced Ovarian Cancer via Laparoscopy Versus Open Surgery: An Analysis of the National Cancer Database
Source: J Clin Med. 2025 Feb 11;14(4):1164. doi: 10.3390/jcm14041164 (PMC11856761; doi:10.3390/jcm14041164)
Supplement: Supplementary file 1 [file jcm-14-01164-s001.zip › jcm-3412619-supplementary.pdf]

Supplementary Material

Table S1: Number of conversions to laparotomy based on minimally invasive approach.

	Overall, N= 681	Laparoscopic, N= 371	Robotic, N= 310	*p* value
Converted, n (%)	57 (8.4)	51 (14)	6 (1.9)	<0.001

Table S2: The distribution of surgical approach per year.

Total	Overall, n=2,412	2010, n= 177	2011, n= 175	2012, n=253	2013, n= 277	2014, n= 305	2015, n=348	2016, n= 304	2017, n= 285	2018, n= 151	2019, n= 137	*p* value
Approach												<0.001
Laparoscopic, n (%)	320 (13)	10 (5.6)	22 (13)	37 (15)	37 (13)	45 (15)	39 (11)	36 (12)	56 (20)	23 (15)	15 (11)	
Robotic, n (%)	304 (13)	11 (6.2)	12 (6.9)	16 (6.3)	28 (10)	32 (10)	53 (15)	48 (16)	41 (14)	28 (19)	35 (26)	
Open, n (%)	1788 (74)	156 (88)	141 (81)	200 (79)	212 (77)	228 (75)	256 (74)	220 (72)	188 (66)	100 (66)	87 (64)	
